# Histological examination of the effect of concentrated growth factor (CGF) on healing outcomes after maxillary sinus floor augmentation surgery

**DOI:** 10.25122/jml-2021-0294

**Published:** 2023-02

**Authors:** Mohammad Ghasemirad, Mohammad-Taghi Chitsazi, Masoumeh Faramarzi, Leila Roshangar, Amirreza Babaloo, Ramtin Chitsazha

**Affiliations:** 1 Department of Periodontics, Faculty of Dentistry, Tabriz University of Medical Sciences, Tabriz, Iran; 2 Dental and Periodontal Research Center, Faculty of Dentistry, Tabriz University of Medical Sciences, Tabriz, Iran; 3 Department of Anatomical Sciences, Stem Cell Research Center, School of Medicine, Tabriz University of Medical Sciences, Tabriz, Iran

**Keywords:** sinus floor augmentation, lateral window sinus lift, CGF

## Abstract

A double-blind clinical trial was conducted to examine the effect of concentrated growth factor (CGF), a new generation of platelet derivatives, on the healing outcome of maxillary sinus floor augmentation during maxillary sinus lift surgery. The study included 9 patients referred to the Tabriz University, Faculty of Dentistry, aged 30-80 years, with bilateral posterior partial edentulous or edentulous maxilla who underwent the procedure using a split-mouth technique. After lifting the Schneiderian membrane, bovine xenograft was randomly applied on one side (for example, left maxillary sinus) and CGF on the other side (for example, right maxillary sinus). Results from alizarin red and hematoxylin-eosin staining methods showed that the percentage of bone formed in the CGF group (112.41±26.34% and 96.16±24.49%, respectively) was significantly higher than in the control group (64.99±24.96% and 60.16±16.39%, respectively) (P<0.05). In addition, after 6 months, the amount of residual graft material in the control group (xenograft) was significantly higher than in the CGF group (P<0.05). These findings demonstrate that the use of CGF during open sinus lift surgery is reliable for the placement of dental implants.

## INTRODUCTION

Recent advances in surgery, implants, and graft materials have enhanced the prognosis of implant treatments in the maxillary posterior area [1]. The lateral window sinus lift technique was first introduced by Tatum in the late 1970s and has been frequently modified since it was published by Boyne in 1980 [2]. To perform this technique, the anatomy of the sinus is first assessed using cone beam computed tomography (CBCT), and a cavity is then prepared in the lateral sinus wall to provide enough bone to place the implant at standard length. For this purpose, the Schneiderian membrane is also detached from the sinus bone walls using appropriate tools and lifted under the sinus membrane, and grafting materials are used to maintain the created space [3].

Various grafting materials are applied for lifting the maxillary sinus floor, including autologous bone, xenografts, mineralized and demineralized bone allografts, and alloplasts [4]. Platelet derivatives such as platelet-rich fibrin (PRF), a rich source of different growth factors, have been gaining attention in sinus augmentation [5,6]. The implant is less likely to survive when PRF is used alone in sinus lift, and simultaneous implant placement is done [7]. Nevertheless, when combined with allografts, PRF showed good results in sinus lift procedures [7].

Compared to PRF, CGF fibrin matrices are larger, denser, and have a more robust network structure, making them more effective in increasing osteogenesis. In addition, CGFs are more cost-effective and can be supplied more quickly, increasing their appeal for use in sinus augmentation procedures [8,9]. Furthermore, CGFs are rich in growth factors, platelet, white blood cells, and stem cells CD34^+^, which increase the probability of regeneration and decrease the risk of infection [10]. CGF releases growth factors such as insulin-like growth factor, vascular endothelial growth factor (VEGF), platelet-derived growth factor (PDGF), and transforming growth factor [11]. CGFs are also able to stimulate the regeneration of bones with osteoporosis [12].

In one study on lifting the sinus floor by osteotomy technique using the CGF and placing short implants among patients with severe maxillary atrophy, Chen *et al*. showed that the height of the alveolar bone decreased after 6 months. However, bone loss occurred within the second 6-month period, which was statistically insignificant [13]. A systematic review by Lokwani *et al*. (2020) also acknowledged the role of CGF in promoting bone formation around implants, either by itself or in combination with allografts or xenografts. The authors concluded that the use of CGF led to an improvement in the quality of bone formation around the implants [14]. This clinical trial study histologically evaluated the effect of CGF on the amount of newly formed bone and the percentage of newly formed bone relative to the former bone. Furthermore, we assessed the amount of residual graft material and the amount of fibrous connective tissue evaluated and compared it to the xenograft material group.

## MATERIAL AND METHODS

This double-blind clinical trial study was conducted using the split-mouth method. 9 patients aged 30-80 with bilateral posterior partial or edentulous maxilla referred to the Tabriz Faculty of Dentistry participated in this research.

The sample size was determined according to Chitsazi MT *et al*. [15,16] by considering the error type one (α = 0.05), power of test 90% and to increase the study validity, 20% was added to the sample size, and 9 patients were finally considered. In each person, one side was randomly selected as the control group (bovine xenograft) and the other as the test group (CGF).

The inclusion criteria were bilateral posterior partial edentulous or edentulous maxilla and height of remaining bone between the alveolar crest and sinus floor less than 5 mm [16]. Pathological signs and symptoms, cystic lesions, acute and chronic inflammatory disease, benign and malignant tumors in the sinus, systemic diseases like uncontrolled diabetes, cardiovascular disease, malignancy, head and neck radiotherapy, autoimmune diseases, use of steroids and immune system suppressing medicines, bisphosphonates, untreated periodontal and periapical diseases, particularly in the sinus floor adjacent teeth, smoking and pregnancy were among the exclusion criteria [16].

**Figure 1:**
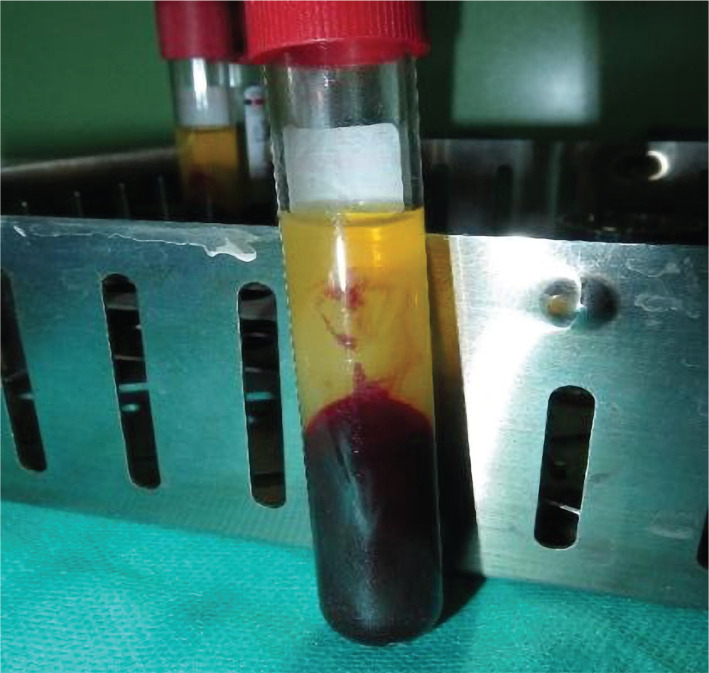
Layers formed in a test tube.

To prepare the CGF, immediately before surgery, intravenous blood was collected into plastic tubes (4-6 tubes) without anticoagulant but containing silicate as a clot activator (each tube 9 mm) and centrifuged using Medifuge (Silfradent Srl, Sofia, Italy) [17]. The program was performed as follows: acceleration 30s, 2800 rpm 2 minutes, 2400 rpm 4 minutes, 2700 rpm 4 minutes, 3000 rpm 3 minutes and 30s speed reduction and then full stop. The whole rotation period was approximately 14 minutes. The whole blood was divided into four linings: (1) serum layer at the top, (2) second layer of buffy coat, (3) growth factor and stem cell layer (CGF), and (4) red blood cell (RBC) at the bottom (Figure 1). The CGF clot was removed from the tube and separated from RBC with a microscopic scissor.

All surgeries were performed by one surgeon. Before injecting the anesthesia, 2% lidocaine, and 1:80,000 epinephrine, patients rinsed their mouth with chlorhexidine gluconate 0.12% for one minute.

**Figure 2:**
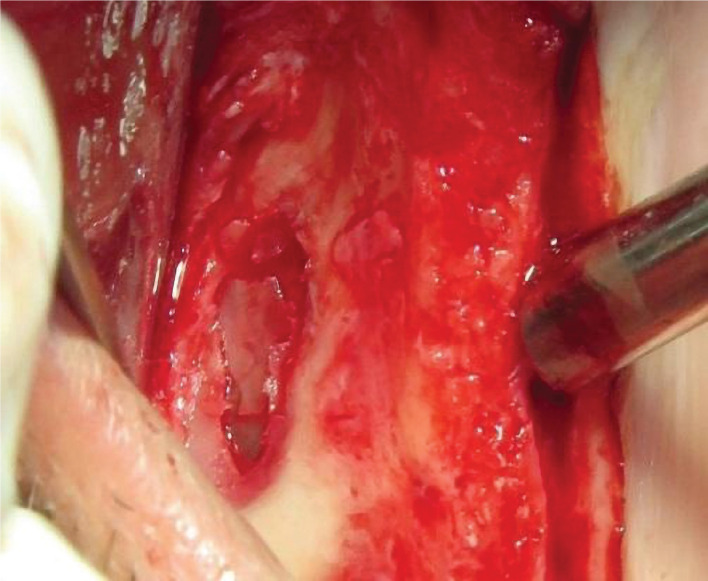
Preparing the bone window in the lateral wall.

Full-thickness crestal incision and, if needed, vertical incision were carried out. To treat the maxillary posterior edentulous area, the lateral window sinus lift technique was applied so that after the reflection of the flap, the outline of the window was determined using the carbide round bur No. 8 in the lateral wall of the sinus bone. Then by the erosion method, the lateral wall window bone was gently removed using the carbide round bur No. 8 and 6, and the Schneiderian membrane was gently elevated (Figure 2).

The Schneiderian membrane (which contains 3 layers: periosteum, connective tissue, and respiratory epithelium) was lifted to prevent the small possible perforations resulting from the sharpness of the edges of xenograft particles in the control group and increase the membrane thickness and to prevent its possible collapse in the intervention group. As the sinus membrane was lifted, a layer of acellular dermal matrix (ADM) (CenoDerm; Tissue Regeneration Corporation, Kish, Iran) with 1-1.4 mm thickness, immersed in sterile saline based on producers recommendations, was placed under the Schneiderian membrane and the grafting substitutes were placed into the cavity [8,18]. Meanwhile, to help prevent the collapse of the Schneiderian membrane into the space made from the sinus lifting, an absorbable collagen sponge (Hemospon; Maquira, Maringa, Brazil) was also placed in the position [18]. After the surgery, amoxicillin/clavulanic acid 500/125 mg was prescribed for patients every 8 hours for 7 days.

**Figure 3:**
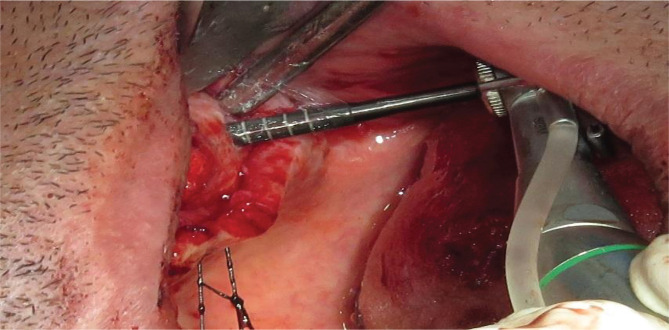
Placing the CGF clots in the sinus cavity.

After lifting the Schneiderian membrane, bovine xenograft was randomly applied on one side (for example, left maxillary sinus) (Bone +B; Nova Teb Pars, Marzanabad, Iran) [19], and CGF on the other side (for example, right maxillary sinus) for all participants (Figure 3).

**Figure 4:**
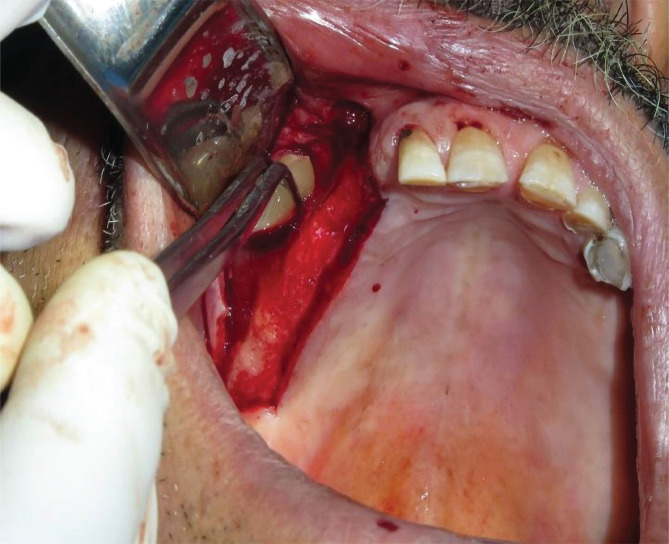
Preparing the biopsy sample after 6 months during the implant placement.

**Figure 5:**
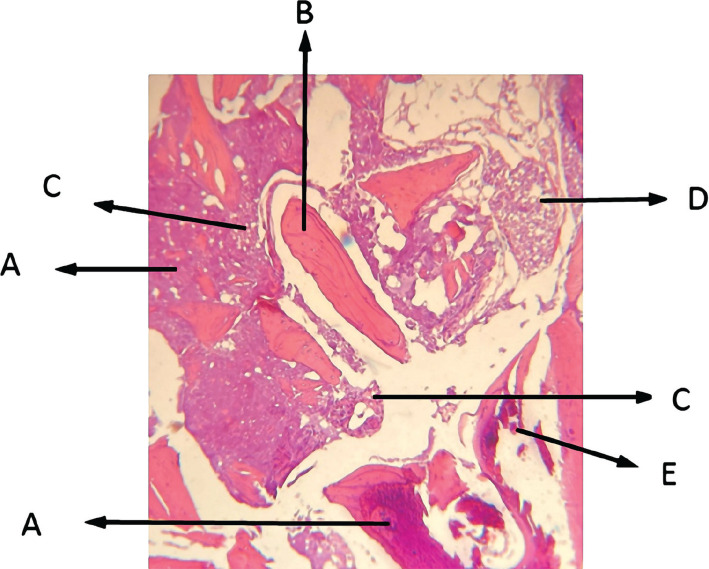
Histological view of sinus augmentation area (H&E staining control group with 100x). A: New bone area with dense bone tissues and newly formed acidophilic bone islands; B: Primary bone of maxillary sinus in acidophilic form with lamellar bone, among which the osteocyte cells were molded; C: Connective tissue with connective tissue cells, collagen fibers, and lymphatic cells. Fibroblasts are seen between the collagen fibers; lipid cells and inflammatory cells like lymphocytes are observable among the collagen fibers; D: Loose connective tissue in the bone marrow, which is full of mesenchymal and lipid cells together with the blood cells; E: Remaining materials.

**Figure 6:**
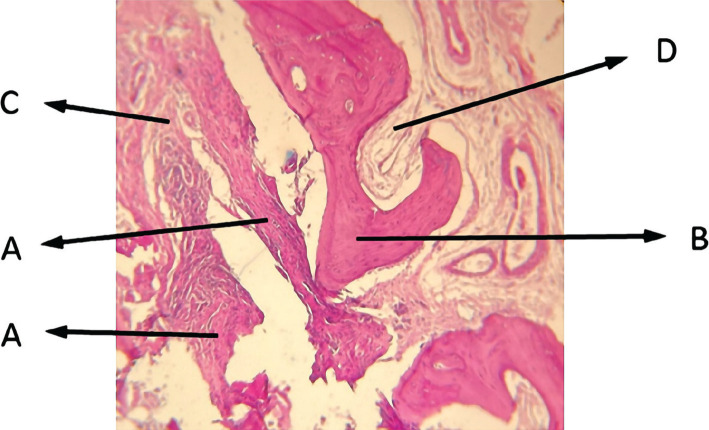
Histological view of sinus augmentation area (H&E staining intervention group with 100x). A: Newly formed bone acidophil area with relatively irregular collagen fibers and osteocyte cells among the collagen fibers. B: Primary bone of maxillary sinus with osteocyte cells inside the clear lacuna among the bone acidophil lamellae. C: Irregular dense connective tissue together with inflammatory cells like lymphocytes and irregular collagen fibers, and fibroblast cells. D: Bone marrow with blood cells inside the mesenchymal tissue.

**Figure 7:**
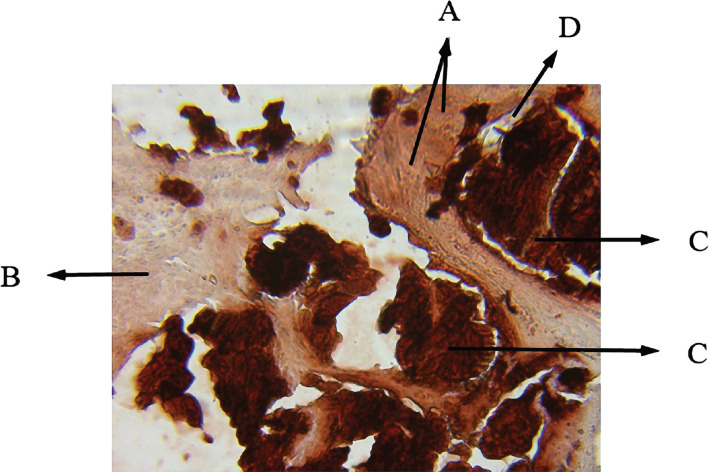
Histological view of sinus augmentation area (alizarin red staining control group with 100x). A: New bone areas in acidophil stain that is differentiable from the previous bone tissue; B: Primary bone of maxillary sinus; C: Remaining xenograft materials are seen between the bone; D: Connective tissue.

**Figure 8:**
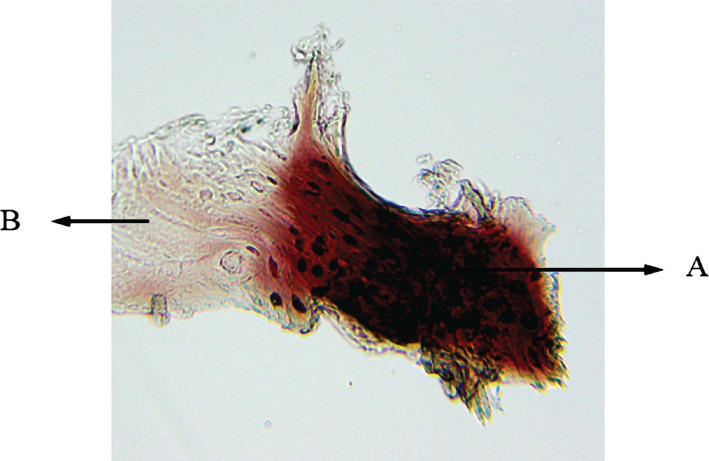
Histological view of sinus augmentation area (alizarin red staining intervention group with 100x). A: New bone areas in acidophil stain that is differentiable from the previous bone tissue; B: Primary bone of maxillary sinus.

**Figure 9:**
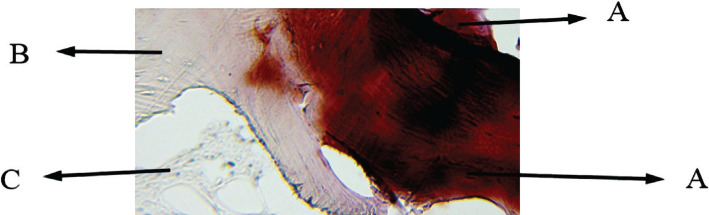
Another histological view of sinus augmentation area (staining intervention group with 100x). A: New bone areas in acidophil stain are differentiable from the previous bone tissue; B: Primary bone of maxillary sinus; C: Connective tissue.

6 months later, CBCT radiography was performed for both groups. Then, the osteotomy area was specified during the surgery using the thin trephine bur with a 2.7 mm internal diameter (Figure 4). The histologic samples were collected, placed in labeled containers filled with a 10% formalin buffer solution, and sent to the medical faculty's histologic laboratory for further examination. Once the tissue passage and routine and specialized staining (hematoxylin-eosin and alizarin red) were performed, microscopic slides were prepared. Parameters were evaluated under a light microscope (BX40, Olympus, Germany). The images were analyzed using Motic Images 2 software (Figures 5–9). The amounts of recently formed living bone, remaining graft material, and formed fibrous connective tissue were analyzed and measured in mm^2^, and the percentage of newly formed bone was reported as the ratio of new bone to former bone [16,20]. The histologist was unaware of the sampled material and the studied groups.

## RESULTS

In this double-blind, split-mouth clinical trial, 18 maxillary sinuses from 9 patients with bilateral posterior partial or edentulous maxilla were examined. Sinus floor elevation was performed on each patient, and CGF was used on one side, while xenograft was used on the other side. The Kolmogorov-Smirnov test indicated that using the alizarin red staining technique, all variables except connective tissue had a normal distribution, and thus parametric tests were used to compare the control and intervention groups. For the connective tissue, a non-parametric test (Wilcoxon) was used. Similarly, all variables in the hematoxylin-eosin histologic technique were normally distributed, and thus parametric tests were applied.

### Alizarin red staining technique

The mean of the formed bone in both groups was significantly higher 6 months after the surgery. The bone area in the control (99.43±37.30) and CGF (95.83±43.76) groups was not significantly different before surgery, and 6 months after the surgery, no significant difference was observed between the control (169.71±87.30) and CGF (197.00±72.59) groups. There was no significant difference between the newly-formed bone area in the control (70.28 ± 52.58) and CGF (101.17 ± 31.73) groups, although the amount of newly-formed bone was higher in the CGF group (Table 1).

**Table 1:** Bone area (mm^2^) before and 6 months after the surgery in control and intervention groups.

		No.	Before the surgery		6 months after the surgery		P-value*	Area of formed bone		P-value*
			Mean	SD	Mean	SD		Mean	SD	
**Alizarin red**	Intervention	9	95.83	43.76	197.00	72.59	<.001	101.17	31.73	<.001
	Control	9	99.43	37.30	169.71	87.30	<.001	70.28	52.58	<.001
**P-value**			0.853		0.461			0.151		
**Hematoxylin eosin**		9	13.01	6.52	26.57	15.04	<.001	13.57	8.66	<.001
		9	13.90	8.92	21.82	13.11	<.001	7.93	4.36	<.001
**P-value**			0.812		0.485			0.107		

P-value: Paired Sample T Test; * P-value: Paired Sample T Test.

### Hematoxylin eosin staining technique

The mean of the formed bone in both groups was significantly higher 6 months after the surgery. The bone area between the control (13.90±8.92) and CGF (13.01±6.52) groups was not significantly different before surgery and 6 months after surgery no significant difference was observed between the control (21.82±13.11) and CGF (26.57±15.04) groups. The area of newly-formed bone in the control and CGF groups was not significantly different, although the amount of newly-formed bone was higher in the CGF group (Table 1).

Evaluating the percentage of formed bone (new bone relative to the former bone) 6 months after surgery using the alizarin red method, the percentage of formed bone in the CGF group (112.41±26.34) was significantly higher than in the control group (64.99±24.96) (P<0.05). Furthermore, in the hematoxylin-eosin method, the percentage of formed bone in CGF (96.16±24.49) was significantly higher than in the control group (60.16±16.39) (P<0.05) (Table 2).

**Table 2:** The percentage of formed bone, remaining material, and connective tissue in control and intervention groups using two staining techniques.

		Alizarin red		Hematoxylin eosin	
		Mean	SD	Mean	SD
**Formed bone**	Intervention	112.41	26.34	96.16	24.49
	Control	64.99	24.96	60.16	16.39
	P-value	.001		.002	
**Remaining material**	Intervention	0.00	0.00	0.00	0.00
	Control	41.66	35.51	0.22	0.16
	P-value	0.003		0.001	
**Connective tissue**	Intervention	2.75	5.09	0.39	0.47
	Control	2.29	4.75	0.54	0.54
	P-value	0.468*		0.155*	

P-value: Paired Sample T Test; * Wilcoxon Test.

There were significant differences in the amount of remaining material in the alizarin red staining technique between the control and CGF groups, being zero in CGF and significantly lower than in the control group with 41.66±35.51 (P<0.05). In the hematoxylin-eosin technique, the amount of remaining material showed a significant difference in both groups, such that it was zero in CGF and significantly lower than in the control group with 0.22±0.6 (P<0.05) (Table 2).

There were no significant differences in the amount of connective tissue between the control (2.29±4.75) and the CGF group (2.75±5.09) in the alizarin red method. Furthermore, there were no significant differences in the amount of connective tissue in the control (0.54±0.54) and the CGF group (0.39±0.47) in the hematoxylin-eosin technique (Table 2).

## DISCUSSION

The present study histologically evaluated the area and percent of newly formed bone in maxillary sinus lift surgery using the lateral window approach and CGF compared to xenograft as graft material. After 6 months of follow-up and using different staining techniques, this study revealed a significant difference in the mean of osteogenesis at the time of using grafting material than before the surgery. Furthermore, in both histological staining techniques, i.e., alizarin red and hematoxylin-eosin, the percentage of newly formed bone in CGF was significantly higher than in the control.

Similarly, the results of both hematoxylin-eosin and alizarin red staining techniques after 6 months revealed that the amount of remaining grafting materials in the control (xenograft) group was significantly higher than in the CGF group. A low value of remaining material in each group indicates the higher speed of graft material absorption. The type of grafting materials and their absorbability affect the percentage of the remaining material.

Shetty *et al*. (2018) described sinus augmentation in the lateral window using CGF and simultaneous implant placement as an appropriate treatment option for the posterior atrophic maxilla. In this study, the mean bone growth in intervention and control (without grafting material) groups was 3.19 mm and 4.47 mm. A statistically significant difference was also observed in bone quality between both groups [21]. Although their assessment was made by radiography, their results are consistent with this study.

In the present study, implants were placed 6 months after the sinus surgery, and sampling for further histological examinations was simultaneously done. A considerable percentage of osteogenesis in both xenograft and CGF groups indicates the usefulness of grafting materials and the formation of new bone for implant placement. The results also indicate the significant effectiveness of CGF on healing outcomes and on increasing bone quality which can be likely attributed to the various growth factors in the dense and rich structure of the CGF fibrin network.

Sohn *et al*., in their study, used CGF as grafting material in sinus augmentation by lateral window approach, and simultaneous implants were placed. After 6 months, they histologically examined 5 biopsy samples taken from the osteotomy of lateral window bone that was put in place, and they reported new active bone formation without inflammation in the maxillary sinus [22].

In an electronic microscopic investigation, CGF showed a thicker and denser fibrin network in a unit than PRF. Basically, CGF is an upgraded version of PRF with a reinforced fibrin matrix and more growth factors [23]. Qin *et al*. demonstrated that CGF could release growth factors for at least 13 days [24]. However, Yu *et al*., in their in vitro study, after 28 days, showed that the level of released growth factors from CGF was increasing, and the most ascending trend occurred within days 14-28 [25]. In this study, the area of CGF use likely follows the trend shown by Yu *et al*.

Adalı *et al*. (2012) placed the implant 6 months after the sinus surgery, and histophotometric examinations showed that the percentage of newly formed bone in allograft along with CGF (on average, 36.41%) was higher than the allograft group (on average, 35.49%), but this difference was not significant [26]. Therefore, it can be said that compared to the combination of CGF and another material, the CGF application by itself is more suitable. On the other hand, the issue of preventing the Schneiderian membrane collapse should be considered when CGF is used by itself. However, in this study, an ADM was first placed under the Schneiderian membrane to help increase its thickness and prevent collapse after sinus lift surgery. Meanwhile, to prevent the collapse of the Schneiderian membrane into the space that was made after sinus membrane elevation, an absorbable collagen sponge was placed in the cavity so no membrane collapse was observed. Similarly, in their study, Adalı *et al*. (2021) evaluated the amount of remaining material in two groups of allograft with CGF and allograft. The percentage of remaining grafting material in the control group (on average 5.80%) was higher than in the intervention group (on average 5.10%) but not statistically significant. They evaluated the height of bone 6 months after the surgery rather than immediately after surgery using CBCT, showing that in the allograft group, decreased height (9.32%) was significantly higher than the allograft with CGF (6.37%) [26]. In other words, CGF was effective in maintaining the new bone.

Tekin *et al*. (2019) evaluated the increased height of bone after a close sinus lift and CGF placement with implant and reported that the results of the intervention group (CGF) were better than the control group (with no grafting material), although not statistically significant [27]. Their results, in contrast to the present one, do not show the significant effect of CGF on osteogenesis.

In their study, Thor *et al*. reported that osteogenesis in the maxillary sinus does not need biomaterials [28]. Riben *et al*. suggested that space maintenance through implant placement leads to blood clot formation, and then the presence of osteoblast cells originating from the periosteum or maxillary spongy bone is responsible for bone formation in this area [29]. In contrast, in their animal study, Kim *et al*. showed that bone formation is very low when no grafting material is used in sinus augmentation [30]. Consistent with Kim *et al*., Sul *et al*. reported that osteogenesis around implants inserted in the maxillary sinus was very low [31].

Regardless of the research mentioned above, a solution should be found after the sinus membrane elevation to keep it up and prevent the membrane collapse, which can be the bone graft materials (e.g., allograft or xenograft particles) or the same technique as in this study, i.e., acellular dermal matrix and collagen sponge.

In this study, there was no significant difference in the amount of formed connective tissue assessed by hematoxylin-eosin and alizarin red staining in both CGF and xenograft groups. In the study by Chitsazi *et al*., no significant difference was seen in the formed connective tissue between two groups, allograft with PRF and allograft with PRF and laser. High values of fibrosis connective tissue compared to the newly-formed bone in every group can indicate the decreased potential or amount of osteogenesis in that group and the formation of fibrosis connective tissue instead of new bone formation [16,32].

In the study by Chitsazi *et al*. (2016), the amount of remaining material in the PRF group with allograft was 29.11, while this value in xenograft in the present study was 41.66. Given the similarity of the research method in both studies, one can probably say that the higher value of the remaining material in the control group in the present study is due to the late absorption property of xenograft against the allograft [16,33].

In a histologic study, Galindo-Moreno *et al*. (2018) examined non-mineralized tissue in the test (xenograft mixed with autogenous bone) group compared to the control (allograft mixed with autogenous bone) group to evaluate the healing outcome after maxillary sinus augmentation. More osteoid lines and vessels and higher cellularity in mm^2^ were reported, indicating higher activity and tissue remodeling in the xenograft group [34]. It is possible that the late absorption of xenograft particles keeps the space for a longer time for the formation of new bone and prevents the collapse of the Schneiderian membrane.

Finally, considering our study and the research presented above, PRP, PRF, and CGF effectively promote new bone formation and achieve improved therapeutic outcomes. However, it should be noted that using these platelet-based compounds entails auxiliary equipment, which incurs additional costs. It is hoped that with advancements in technology, the cost of equipment and the duration of use in clinical practice will be reduced in the future. In light of the significance of this topic, it is recommended that future studies be conducted with larger sample sizes and longer follow-up periods for more comprehensive and thorough evaluations. For subsequent studies, it is suggested to examine the effect of CGF in combination with allograft or xenograft for the long term as well as the effect of CGF application on the success and stability of placed implants.

## CONCLUSION

The results of the histologic evaluation suggest that open sinus lift surgery using CGF can be a reliable option for implant placement. Both alizarin red and hematoxylin-eosin staining techniques showed that the percentage of newly formed bone in the CGF group was significantly higher than in the control (xenograft) group after 6 months. The remaining material in the control group was significantly higher than in the intervention. On the other hand, the amount of connective tissue between both groups was the same.

## Personal thanks

The authors wish to thank the Vice Chancellor for Research of Tabriz Dental Faculty for their support and express their gratitude to Ms. Afsaneh Ghalami, the person in charge of the nursery part of the periodontics department in Tabriz Faculty of Dentistry, because of her help during the blood-sampling stages.
